# Positive-Unlabeled Learning for inferring drug interactions based on heterogeneous attributes

**DOI:** 10.1186/s12859-017-1546-7

**Published:** 2017-03-01

**Authors:** Pathima Nusrath Hameed, Karin Verspoor, Snezana Kusljic, Saman Halgamuge

**Affiliations:** 1 Department of Mechanical Engineering, University of Melbourne, Parkville, Melbourne, 3010 Australia; 2 Data61, Victoria Research Lab, West Melbourne, 3003 Australia; 3 Department of Computer Science, University of Ruhuna, Matara, 81000 Sri Lanka; 4 Department of Computing and Information Systems, University of Melbourne, Parkville, Melbourne, 3010 Australia; 5 Department of Nursing, University of Melbourne, Parkville, Melbourne, 3010 Australia; 6 The Florey Institute of Neuroscience and Mental Health, University of Melbourne, Parkville, Melbourne, 3010 Australia; 7 Research School of Engineering, College of Engineering and Computer Science, The Australian National University, Canberra, 2601 ACT Australia

**Keywords:** Drug-drug interaction, Growing self organizing map (GSOM), Pairwise drug similarity, CYP isoforms, PU learning

## Abstract

**Background:**

Investigating and understanding drug-drug interactions (DDIs) is important in improving the effectiveness of clinical care. DDIs can occur when two or more drugs are administered together. Experimentally based DDI detection methods require a large cost and time. Hence, there is a great interest in developing efficient and useful computational methods for inferring potential DDIs. Standard binary classifiers require both positives and negatives for training. In a DDI context, drug pairs that are known to interact can serve as positives for predictive methods. But, the negatives or drug pairs that have been confirmed to have no interaction are scarce. To address this lack of negatives, we introduce a Positive-Unlabeled Learning method for inferring potential DDIs.

**Results:**

The proposed method consists of three steps: i) application of Growing Self Organizing Maps to infer negatives from the unlabeled dataset; ii) using a pairwise similarity function to quantify the overlap between individual features of drugs and iii) using support vector machine classifier for inferring DDIs. We obtained 6036 DDIs from DrugBank database. Using the proposed approach, we inferred 589 drug pairs that are likely to not interact with each other; these drug pairs are used as representative data for the negative class in binary classification for DDI prediction. Moreover, we classify the predicted DDIs as Cytochrome P450 (CYP) enzyme-Dependent and CYP-Independent interactions invoking their locations on the Growing Self Organizing Map, due to the particular importance of these enzymes in clinically significant interaction effects. Further, we provide a case study on three predicted CYP-Dependent DDIs to evaluate the clinical relevance of this study.

**Conclusion:**

Our proposed approach showed an absolute improvement in F1-score of 14 and 38% in comparison to the method that randomly selects unlabeled data points as likely negatives, depending on the choice of similarity function. We inferred 5300 possible CYP-Dependent DDIs and 592 CYP-Independent DDIs with the highest posterior probabilities. Our discoveries can be used to improve clinical care as well as the research outcomes of drug development.

**Electronic supplementary material:**

The online version of this article (doi:10.1186/s12859-017-1546-7) contains supplementary material, which is available to authorized users.

## Background

Drug interactions refer to the modification of action of one drug caused by the action of another drug. Thus, drug-drug interactions (DDIs) occur when two or more drugs are administered together. DDIs may abolish, diminish or potentiate the effect of the drugs involved. Some DDIs can also lead to fatalities if an inappropriate drug combination has been chosen [1]. Various factors can affect drug interactions, including binding to plasma proteins, binding to tissues and extravascular sites, activity of the liver enzyme system, and intake of certain food groups [2, 3]. Investigating and understanding new DDIs is crucial in improving health care and patient outcomes.

There are a large number of drugs available on the market, but knowledge of DDIs is incomplete, triggering interest in investigating new DDIs. DrugBank is an online database that provides biochemical and pharmacological information about drugs, their mechanisms and their targets [4]. The most recent release (DrugBank 4.0) contains over 335 thousand biochemical and other DDIs, reflecting the great interest in investigating DDIs in recent years. However, this represents only <1% of the possible drug pairs for the 8206 drugs listed on DrugBank [5], suggesting that many more DDIs have been left undiscovered.

Performing experimental trials for a large number of drug pairs is not realistic in terms of cost and time. Due to animal welfare considerations, an animal-based testing process is also problematic. During the last decade, machine learning [6–11] and statistical models [12, 13] including integration of text mining [14] have gained popularity for inferring DDIs. Even though initial approaches focused on utilizing chemical space properties to compare drug characteristics, heterogeneous data integration has been recognized to be significant in developing reliable computational models to infer drug interactions [1, 6, 9, 10]. Therefore, we consider a range of drug characteristics in our study.

DrugBank can be considered as the gold standard database for understanding and learning DDIs, there is no gold standard database for non-interacting DDIs. To train binary classifiers, such negatives are needed. Therefore, in this research, we propose to address DDI prediction with a Positive-Unlabeled Learning (PUL) approach and to demonstrate a suitable method for identifying potential negatives to learn a standard binary classifier. The objective is to identify potential non-interacting DDIs from unlabeled data to allow them to be treated as negatives. Learning from positives and unlabeled data has seen successful application in the medical diagnosis context, as well as for uncertain data, streaming, text, and Web [15]. In such applications, the available labeled items are only positive examples while the negative class data is unknown or unavailable and therefore may include both positives and negatives as a mixture; this characteristic matches well to the situation with DDIs.

Our investigation makes three primary contributions: i) we propose a PUL approach based on Growing Self Organizing Map (GSOM) [16] clustering to infer DDIs; ii) we propose a new pairwise similarity function to quantify the overlap of drug features along several dimensions; and iii) we classify the predicted DDIs as Cytochrome P450 (CYP)-Dependent and CYP-Independent interactions by invoking their locations in GSOM.

### Positive-unlabeled learning

**Fig. 1:**
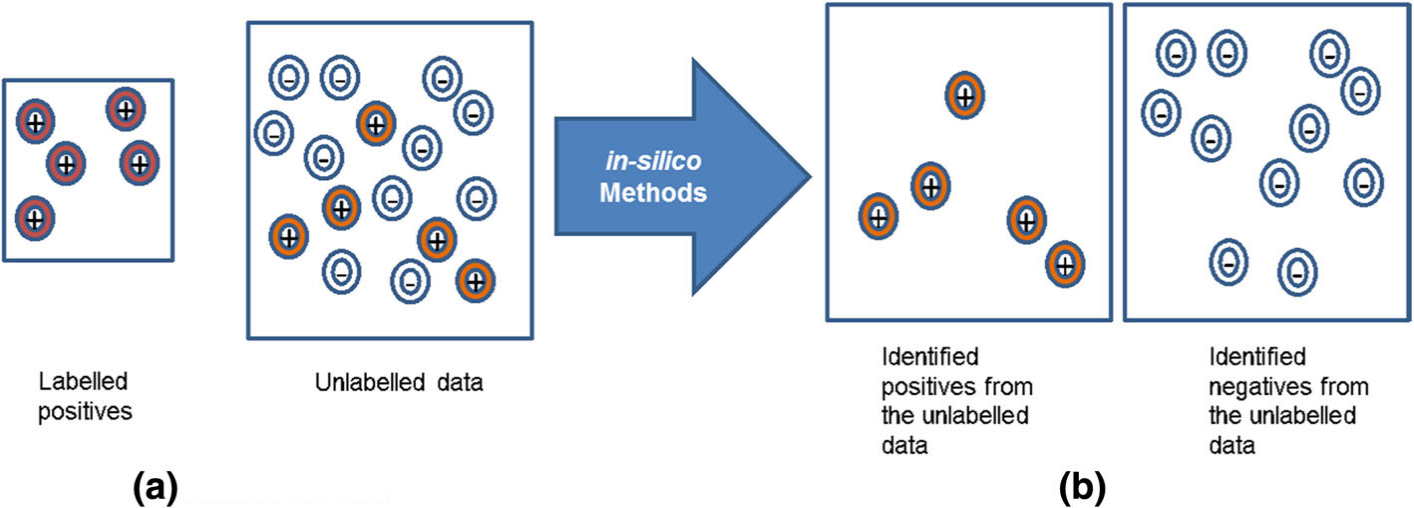
This diagram illustrates the main idea behind Positive-Unlabeled Learning. **a** Available data. **b** Goal

In some applications, only positive examples are known and labeled while the unlabeled data may contain both negatives as well as unlabeled positives. Extracting positives from the unlabeled data is a useful task, and this is more challenging compared to traditional supervised classification problems where clear negatives exist. Figure 1 illustrates the main idea behind Positive-Unlabeled Learning (PUL) scenario, where only positive examples are labeled while the unlabeled data contain both negatives and unlabeled positives. The ultimate goal is to identify useful positive examples from the unlabeled data.

Computational methods such as one class learning and semi-supervised approaches have been proposed to address PUL problems [17, 18]. Semi-supervised learning involves a two step learning strategy where the initial step employs random initialization of negatives from the unlabeled data the subsequent step applies iterative learning to refine the predictions. An integrated Naive Bayes and iterative Support Vector Machine (SVM) approach has been considered as the baseline approach in PUL. The Naive Bayes classifier is used in the initial classification to identify a possible negative set and then the iterative learning uses the SVM. However, Rocchio algorithm may outperform Naive Bayes [17]. Rocchio algorithm is a linear classifier based on cosine similarity. However, it is difficult to extend for a multi class environment. Also, it may extract false negatives if the decision boundary is non-linear. Further purification of Rocchio output using k-means clustering is recommended to identify false positives and false negatives [17].

One Class Support Vector Machine (OCSVM) [19] classifier has been utilized in order to extract the positive class instances from the unlabeled data. In contrast to binary/multi class SVM, OCSVM defines a hypersphere on higher dimensional space. One Class Logistic Regression has been proposed as a strategy to overcome the limitations of the traditional OCSVM [20]. Revealing the poor performance of the trained classifiers using uncertain data, they estimate probabilities for the final predictions using One Class Logistic Regression. However, not using a negative set limits the analysis and evaluation. On the other hand, Ren et al. [21] suggested the importance of using binary classification over OCSVM to achieve better predictive performance. They suggest weighted SVM to identify likely negatives from the unlabeled data. Their results reveal Recall of 71% though Precision and F-Score are relatively low.

In the PUL context, clustering is used as an approach to logically separate unlabeled data [17]. It is used as a pre-step for binary classification to identifying likely negatives from the unlabeled data. Then, the binary classification can be employed to further refine the identified positives using initially known positives and identified likely negatives. Unlike classification, clustering is applied on the inputs concealing their labels and may identify new patterns based on the input characteristics. Clustering algorithms like K-means, and Self Organizing Map (SOM) require the number of possible clusters to be specified in advance. In medical and clinical applications, separating the unlabeled data into two groups is not appropriate as they can be divided into more than two groups based on finer characteristics. Similarly, there are different and various types of DDIs and knowing them is important when performing unsupervised learning using K-means and SOM. Therefore, clustering DDIs is a challenging task. In this paper, we propose GSOM [16] as a suitable clustering approach to cluster drug pairs to enable inferring negatives from the unlabeled dataset. The main advantage of GSOM is automatic detection of the number of subgroups/nodes. Its high dimensionality feature reduction and topology preserving nature are also useful characteristics.

In the related work, randomly selected instances from the unlabeled data has been used as neutral DDIs (negatives) [1, 6–8, 22–24]. We treat this existing method as our ‘Baseline’. This approach may introduce noisy data, resulting in a lack of distinction between positives and negatives. However, the overall performance of the final prediction relies on the feature representation and the training sample as well. We believe that the clinical relevance of the predicted DDIs can be improved employing a representative training sample with comprehensive heterogeneous properties.

### Similarity based DDI prediction

Computational approaches to DDI prediction [1, 6, 8–10, 22, 25] assume that drug pairs sharing similar characteristics (chemical, phenotype, biological, therapeutic, etc.) are more likely to share the same drug interactions. Cheng et al. [7] and Vilar et al. [8] employed chemical properties in predicting new DDIs. However, their methods showed poor sensitivity. Drug interactions like physiological properties cannot be predicted by chemical properties alone, since drugs undergo complicated metabolic transformations and other pharmacokinetic transformations as they are metabolized and physiologically distributed [26, 27].

Recent studies have emphasized the relevance of integration of heterogeneous characteristics, including chemical, phenotype, biological, and therapeutic features, for establishing pairwise drug similarity [1, 6, 9, 10]. Vilar et al. [8] investigated the impact of molecular structural similarities for DDI predictions and obtained performance values of 0.68, 0.96, and 0.26 of sensitivity, specificity, and precision, respectively. Later, Vilar et al. [9] published a new protocol for inferring DDIs integrating 2D and 3D molecular structural, target, and side-effect similarities. In their study, the drug profiles were combined by means of linear algebraic concepts through matrix manipulations. Moreover, they observed higher DDI predictive performance when heterogeneous features were integrated using Principal Component Analysis and the highest individual predictive performance was observed when using Interaction Profile Fingerprints. Similarly, Zitnik and Zupan [13] proposed a probabilistic model using collective matrix factorization to depict the pairwise relations in pharmacological networks. They demonstrated Area Under ROC Curve (AUC) of 0.924 using 10-fold cross validation for predicting DDIs which were found in DrugBank. But, their method can focus on only one particular data type at a time.

Cheng et al. [7] used chemical sub-structural properties in predicting five major CYP isoforms integrating various base classifiers. They constructed combined classifiers for each of the isoforms of interest, obtaining overall predictive accuracy between 72.3 and 83.7%. However, this single-isoform modeling approach does not scale well to large numbers of drugs. Realizing the value of heterogeneity for achieving useful DDI prediction, Gottlieb et al. [6] employed seven heterogeneous characteristics based on chemical, ligand, side effect, annotation, sequence, closeness in a protein-protein interaction network, and Gene Ontology through which they constructed 49 classification features. As a result of deep analysis of CYP-based DDIs, they observed AUC of 0.93 and 0.96 for CYP-Related DDIs and non-CYP-Related DDIs, respectively. They employed binary classification by randomly selecting examples from the unlabeled data to represent the negative class, which may introduce noise, resulting in a lack of distinction between interacting and non-interacting classes in the training set. In general, the outcome of the final predictions rely on the characteristics of the training sample. Thus, selecting a representative training sample is also beneficial in improving predictive performance.

Liu et al. [22] provides a comprehensive analysis of overall contextual similarities to predict plausible DDIs. They employed chemical interactions comprising the overall structural activities and reaction-based similarities. Overall pairwise target similarity is captured analyzing the interactions of target proteins. Pathway analysis was also carried out to capture the related pathways of drugs and their functions. Moreover, a minimum redundancy maximum relevance approach and incremental feature selection were used for dimensionality reduction and to remove redundant features. They achieved specificity ranging between 0.89 and 0.97, and sensitivity ranging between 0.13 and 0.71. In DDI prediction, positives are more important than negatives due to physical consequences of the effect. To emphasize positive interaction prediction, we believe that the measures like sensitivity, precision, and f-score are more important than specificity.

Tanimoto Coefficient is a variant of Jaccard Index which is widely used in quantifying the overall context similarity between drugs. Vilar et al. [9] proposed a similarity metric to quantify the overall overlap between any two drugs. Huang et al. [28] defined a metric to compute the tightness/strength of target centric drugs, acknowledging the impact of protein-protein interaction networks in identifying new DDIs. Similarly, other existing similarity measures provide overall context similarity to compare any two drugs. However, two drug pairs can share the same overall similarity index though their individual features are different. Hence, a detailed similarity metric is also beneficial to represent the pairwise drug similarities. Our proposed similarity metric can be used to summarize any type of drug data associations. Moreover, our results demonstrate the importance of the proposed pairwise similarity measure in obtaining better performance on DDI predictions.

## Methods

### Drug data

#### Drug characterization

We obtained four different independent sources of drug associations following the work of Wang et al. [23]; drug-chemical [29], drug-therapeutic [30], drug-protein [29], and drug-phenotype [31] associations. These associations are represented as binary relationships where ‘1’ represents a known interaction (labeled) and ‘0’ represents an unknown interaction (unlabeled). Each of the drugs has 881, 719, 775, and 1385 dimensions in chemical, therapeutic, protein, and side effect characteristic profiles, respectively. The four independent sources have 548 drugs in common. We calculate pairwise drug similarities using Jaccard Index (JI) and a proposed pairwise similarity function (see “The proposed similarity feature representation” section). JI-based pairwise similarity measure was applied to produce four pairwise drug similarity features (for each type of drug association) while the proposed pairwise drug similarity function led to produce 3760 (881+719+775+1385) pairwise drug similarity features (for each drug in each dimension). Further, we observed 69,600, 2284, 1902, and 41,008 associations between the 548 drugs and 881 chemical features, 719 diseases, 775 proteins, and 1385 side effects, respectively.

#### Drug-drug interactions

DrugBank is a comprehensive database containing extensive biochemical and pharmacological information about drugs, their mechanisms and their targets. It uses data on drug-target, drug-enzyme and drug-transporter associations to provide insight on DDIs [4]. Moreover, it includes drug interaction information from several external sources [32] such as Physician’s Desk Reference [33], e-Therapeutics [34], Medicines Complete [35], Epocrates RX [36], and Drugs.com [37].

We obtained 6036 unique DDIs for the 548 drugs of interest via ‘DrugBank - Interax Drug Interaction Lookup’ [38] (Note: these known DDIs will be served as positives for machine learning). Also, we noticed 994 Cytochrome P450 (CYP)-Dependent DDIs out of 6036 DDIs. The 6036 DDIs span only 451 unique drugs out of the selected 548 drugs. There are 149,878 (548*548-548) possible unique drug pairs for these selected 548 drugs. Hence, the known (labeled) DDIs to unlabeled DDIs ratio is approximately 1 to 24 (1:24).

### Proposed methods

**Fig. 2:**
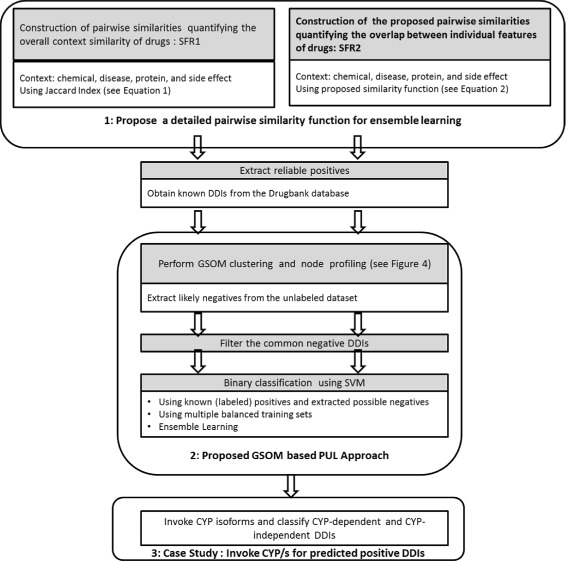
This diagram illustrates the proposed methodology and our three main contributions for inferring DDIs, integrating Similarity Feature Representation1 (SFR1) and Similarity Feature Representation2 (SFR2)

Posing the overall DDI prediction task as a PUL task, we propose a two phase learning strategy integrating GSOM and SVM. Moreover, we propose an ensemble learning approach for DDI prediction based on two different pairwise similarity metrics; we build a classifier using each metric separately and then combine their individual predictions to obtain a final prediction. Figure 2 illustrates the main steps of the proposed approach for inferring DDIs. We emphasize the importance of quantifying the overlap of individual properties as well as the overall context similarity for inferring potential DDIs.

First, we employ the GSOM clustering algorithm to cluster drug pairs based on given chemical, disease, protein, and side effect (pairwise) similarities. We believe that non interacting drug pairs are likely to contain pairwise similarities that are different from the interacting drug profiles. We therefore label GSOM nodes as positives or negatives based on the scattered pattern of known labeled positives on GSOM (see “GSOM-based positive-unlabeled learning approach” section). As a result, likely negatives can be inferred from the unlabeled data for training purposes. Second, SVM is employed for binary classification. Finally, we perform a further analysis on the predicted DDIs considering CYP isoforms (CYP1A2, CYP2C9, CYP2C19, CYP2D6, and CYP3A4). Specifically we identify CYP isoforms for the predicted CYP-Dependent DDIs by revisiting their node on the GSOM, as explained in “Case study: clinical implications” section.

For the 548 selected drugs (see “Drug characterization” section), the 6036 known DDIs correspond to approximately 4% of possible drug pairs (149,878). To the best of our knowledge, there is no gold standard database representing drug pairs that do not interact with each other. Therefore, we pose this problem as a PUL problem. We suggest and demonstrate our proposed GSOM-based PUL approach as a suitable method for inferring DDIs. We select GSOM over other clustering methods due to its automatic detection of the number of relevant clusters; this is not a fixed parameter that needs to be set.

#### GSOM-based positive-unlabeled learning approach

Clustering enables grouping of drug pairs based on their similarity, independently of any labels. It can be used to distinguish drug pairs as interacting and non-interacting DDIs, by identifying pairs that have similar characteristics to (cluster with) a known DDI, and pairs that do not cluster with any known DDI, respectively. We propose GSOM [16] as a suitable approach to cluster drug pairs. GSOM is an extended version of the conventional SOM [39]. A GSOM consists of nodes where each node contains at least one input. This clustering algorithm assigns each of the instances to a node on GSOM map. It uses the same weight adaptation and neighborhood kernel learning as SOM. Further analysis can also be inferred after a map is produced. GSOM’s topological preserving nature is useful in grouping neighboring nodes where necessary.

GSOM’s automatic detection of the number of clusters is useful in this context as we have no prior knowledge of the number of DDI types. GSOM can cluster drug pairs based on their given characteristics and is capable of handling high dimensional data. Since we are unaware of the exact number of DDI types, GSOM enables sub-grouping these drug pairs without any prior knowledge of number of clusters. The size of the map can be controlled by the growth threshold which is inversely proportional to the Spread Factor and it is shown in the equation below:

1 $$ \begin{aligned} Growth\ Threshold=-D*ln(Spread\ Factor) \\ \text{where}\, D \, \text{is the dimensionality of the input data.}  \end{aligned}  $$

Accordingly, the number of growing nodes can be controlled by its Spread Factor, ranging between 0 and 1 ((0,1]). A higher Spread Factor can produce a larger GSOM with a higher number of nodes. In this study, we employ the GSOM implementation of Chan et al. [40] as it provides visual aids for cluster analysis.

In this study, GSOM is used to infer negatives from the unlabeled data. We propose a node profiling algorithm to profile each of the GSOM nodes as a ‘positive’ /‘negative’/‘ambiguous’ node, based on the positive proportion of the inputs clustered within the node. We define Positive Proportion as:

2 $$ {\begin{aligned} &Positive\, Proportion\\ &\,=\, \frac{number\, of\, labeled\, positive\, instances\, in\, the\, GSOM\, node} {total\, number\, of\, instances\, in\, the\, GSOM\, node} \end{aligned}}  $$

**Fig. 3:**
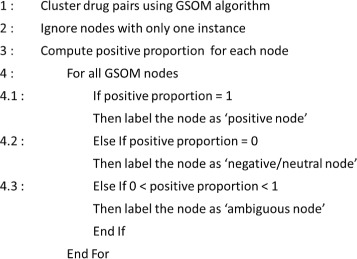
Pseudo-code for profiling GSOM nodes as ‘positive/negative/ambiguous’ node

The nodes with 100% unlabeled instances are considered as ‘negative nodes’. The nodes with 100% positive instances are considered as ‘positive nodes’ and the nodes with both positives and unlabeled instances are considered as ‘ambiguous nodes’. The instances at ‘negative nodes’ are inferred as negatives. Hence, the DDIs collected from DrugBank and the inferred negatives are served as positives and negatives, respectively, for learning the binary classifier whereas the unlabeled instances at ‘ambiguous nodes’ are considered for DDI prediction. The algorithm in Fig. 3 also illustrates the process of profiling GSOM nodes as positive/negative/ambiguous for PUL.

#### Pairwise drug feature representation

##### Overall context similarity using jaccard index

In related DDI prediction tasks, pairwise drug similarities have been derived by quantifying the overall similarity of drug features such as chemical, disease, protein, and side effect [1, 6, 8, 9]. Jaccard Index (JI) based similarity representation is widely used in this context. JI performs a bitwise comparison and computes an overall similarity value as explained in the following equation:

3 $$\begin{array}{*{20}l} JI(Drug_{1},Drug_{2})=\frac{\sum_{i}f_{i} (Drug_{1})\cap f_{i}(Drug_{2})}{\sum_{i}f_{i}(Drug_{1})\cup f_{i}(Drug_{2})} \end{array} $$

where *i* is the index of feature vector, *f*.

**Fig. 4:**
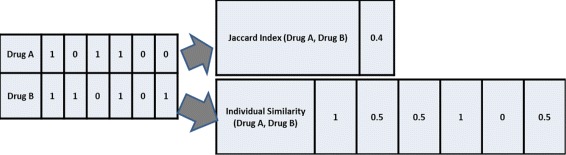
Example of deriving similarity metrics for drug association. Jaccard Index is the frequently used approach while Individual Similarity function is the proposed function

For instance, when drug-chemical associations are represented using 881 chemical sub-structures, the pairwise drug chemical similarity can be mapped into a single similarity value using JI (see Fig. 4). This, JI-based pairwise drug similarity feature representation quantifies the overall similarity of any two drugs based on one set of features. Here, we consider chemical, target-protein, disease and side effect features separately, producing four JI similarity values. This results in Similarity Feature Representation1 (SFR1), which concatenates the four individual JI values into a single feature vector. Each training vector is associated with a label indicating the DDI status of the drug pair; the DDIs derived from DrugBank were considered as positive examples.

##### The proposed similarity feature representation

Reflecting the importance of comparing two drug pairs in terms of their overlap on individual features, we propose a new similarity representation (Similarity Feature Representation2, SFR2), based on the following equation:

4 $$ {\begin{aligned} &Individual~Similarity_{i} (Drug_{1},Drug_{2})\\&\quad=Average(f_{i}(Drug_{1}),f_{i}(Drug_{2})) \end{aligned}}  $$

where *i* is the index of the *i*th feature in the feature vector, *f*.

This captures the shared properties of the drugs, and leads to generate one similarity value per feature; for our data means 881, 719, 775, and 1385 chemical similarity values, disease similarity values, protein similarity values, and side effect similarity values, respectively. Therefore, the proposed SFR2 includes 3760 (881+719+775+1385) similarity features.

As explained in “Drug characterization” section, the initial drug features in our data are binary associations. Therefore, the values on SFR2 include only three values; ‘0’, ‘0.5’, and ‘1’. ‘0’ denotes when a particular feature is absent in both of the drugs, ‘0.5’ denotes when a particular feature is present in only one of the drugs, and ‘1’ denotes when a particular property is present in both of the drugs. As for SFR1, each training vector is associated with a label indicating the DDI status of the corresponding drug pair, obtained from the DrugBank database. Figure 4 illustrates an example on deriving similarity metrics for binary drug-feature representation. Because SFR1 produces a single summary similarity value, two pairs of drugs can have same JI similarity value although their underlying characteristics are entirely distinct. In contrast, SFR2 measures similarity at a granular feature level. Therefore, we suggest aggregating the final results of SFR1 and SFR2.

#### Application to DDI prediction

The Support Vector Machine (SVM) learning method is frequently used in inferring DDIs as well as in the PUL context [1, 17, 21]. Therefore, we employ SVM as the classifier for the supervised learning task. We consider identifying likely neutral DDIs as an important step towards obtaining reliable prediction, as they can serve as negatives for binary classifiers. For the proposed approach, the DDIs obtained from DrugBank database are served as the positives while the neutral DDIs inferred by the GSOM step (see “GSOM-based positive-unlabeled learning approach” section) are served as negatives. We then train a classifier with a training set of known positives and these inferred negatives.

Ensemble Learning is the popular way of combining multiple base classifiers and aggregating outputs to a single meta classifier. For the learning step, we apply ensemble learning with two classifiers employing each of the two similarity functions; SFR1 and SFR2.

We employ probabilistic outputs for Support Vector Machines to compute the posterior probabilities of the classification output following Platt [41]. Platt proposed an approach to calculate the posterior probability by fitting a sigmoid function after building the SVM. It is shown in the equations below:

5 $$ \begin{aligned} P(y=1|f)=\frac{1}{1+exp(Af+B)} \end{aligned}  $$

where the parameters A and B are fitted using maximum likelihood estimation from a training set (*f*_*i*_,*y*_*i*_).

Consequently, we estimate posterior probabilities for all SVM classifier outputs and we average the final probabilities to make final predictions.

Balanced training sets are frequently used in machine learning based DDI predictions [1, 6]. Here, we use multiple balanced training sets to strengthen the ensemble learning approach, through which we obtain an average final probability. This may aid to reduce the variance of the final outputs.

##### Baseline method

In computational DDI prediction, researchers employ binary classification as a step towards inferring drug interactions. Since there is no certain dataset representing neutral DDIs, a baseline method that randomly selects examples from the unlabeled data has been used to identify negative cases [1, 6–8, 22–24]. In this Baseline approach, known positives and randomly selected unlabeled data are used as inputs for the binary classifier.

The explicit steps of this approach is shown below:

1. Initial Data: Generate unique drug pairs for the drugs of interest
2. Collect known DDIs from reliable sources (ex: DrugBank)
3. Label known DDIs (2) as positives in the initial data (1)
4. Randomly select an equivalent number of examples from the unlabeled data (not part of the positive set) to the number of positive cases (3)
5. Label drug pairs (4) as negatives in the initial data (1)
6. Employ binary classification using positives (3) and negatives (5) for DDI prediction

##### Evaluation metrics

It is important to note that there is no certain dataset for the neutral-DDIs (negatives). Therefore, evaluating DDI prediction as a binary classification is a challenging task. We use GSOM-based PUL approach to infer DDIs. Then, F1-score is used to evaluate and compare the performance of the proposed methods. Since our ultimate goal is to predict plausible positives, F1-score is selected to express the overall performance of classifying positive and neutral DDIs [42]. F1-score combines both Precision and Recall metrics as shown in the following equations:

6 $$\begin{array}{*{20}l} Precision & = \frac{True\, Positives}{True\, Positives+False\, Positives} \end{array} $$

7 $$\begin{array}{*{20}l} Recall &= \frac{True\, Positives}{True\, Positives+False\, Negatives} \end{array} $$

8 $$\begin{array}{*{20}l} F1-score &= \frac{2*Precision*Recall}{Precision+Recall} \end{array} $$

## Results

In the proposed PUL approach, we employed known positives and negatives inferred by GSOM from the unlabeled data. We compared the performance of the proposed GSOM-based PUL approach against Baseline and OCSVM [19]. We employed Baseline using known positives and randomly selected negatives from the unlabeled data while OCSVM is employed using known positives only.

In Additional file 1, we demonstrate the improved performance of the proposed GSOM-based PUL approach as compared to Baseline and OCSVM using adapted benchmark data (breast cancer and iris data). The original benchmark datasets are not directly compatible with DDI data as they are fully described with labels where clear negatives exist. Therefore, we modified the labels of these two benchmark datasets to resemble the Positive Unlabeled Learning scenario. We assessed the impact of the proportion of unreliable negatives (unlabeled positives) in the training data towards final prediction; and we defined this proportion as Unlabeled Positive Proportion (UPP). The results on the adapted benchmark datasets (see Additional file 1) suggest that the proposed GSOM clustering as a suitable approach for inferring negatives from unlabeled data. The proposed GSOM-based PUL approach outperforms Baseline and OCSVM. Moreover, the results on the adapted benchmark datasets suggest that the proposed method is most beneficial when the UPP is below 50% (see Additional file 1 for further details).

In this section, we demonstrate the results for inferring DDIs in relation to the proposed pairwise drug similarity and the proposed PUL concept. The results on DDI classification also evidence that the proposed methods can be generalized well to more complex DDI prediction task.

### Inferring plausible DDIs

#### Pairwise drug similarity functions

SFR1 captures the similarity based on JI (see Eq. 3) which is the quantification of four overall context similarities for chemical, therapeutic, protein, and side effect features. We employ SFR2 to quantify the overlap of individual properties between drugs (see Eq. 4) which leads to 3760 features: concatenating 881, 719, 775, and 1385 features in chemical, therapeutic, protein, and side effect characteristic profiles, respectively. It is possible that SFR2 is a sparse vector as some of the features are unique. We therefore employed Principal Component Analysis (PCA) for data compression. PCA is one of the popular methods for dimensionality reduction. It produces a new feature vector capturing maximum variance of the original data. We obtained 228 PCs capturing 90% of maximum variance; these components were used in generating results for SFR2.

#### GSOM-based PUL approach for DDI prediction

In our GSOM-based PUL approach, we employed GSOM clustering to infer negatives from the unlabeled sample. (In Baseline, the known 6036 DDIs were considered as positives while randomly selected 6036 drug pairs from the unlabeled samples were considered as negatives. OCSVM is employed using known 6036 DDIs only.)

##### GSOM clustering using SFR1:

In GSOM, the number of growing nodes can be controlled by altering Spread Factor which varies between 0 and 1 ((0,1]). Higher Spread Factors generate larger maps with relatively higher number of nodes. Also, larger GSOM produces higher coherence within the nodes. We used Average Within Cluster Distance (AWCD) to study the effect of varying Spread Factor within GSOMs; as shown in the Eq. (9).

9 $$ \begin{aligned} AWCD=\frac{\sum_{i=1}^{n}\sum_{j=1}^{m}r_{ij}(f_{j}-\bar{w_{i}})^{2}}{n} \end{aligned}  $$

where $\bar {w}$ is the weight vector of the winning node, *f* is the weight vector of the input, *n* is the number of nodes in the GSOM, *m* is the number of inputs in each node and *r*_*ij*_ is 1 if input *i* ∈ node *j* while *r*_*ij*_ is 0 if input *i* ∉ node *j*.

**Fig. 5:**
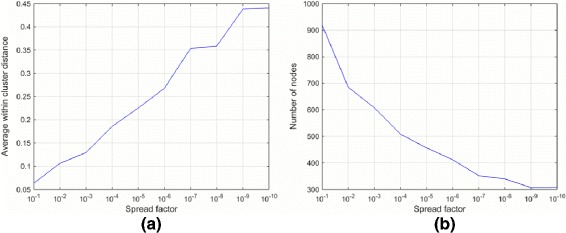
**a** The average within cluster distance (AWCD) using Similarity Feature Representation 1 and (**b**) Number of GSOM nodes variation for Similarity Feature Representation 1

In Fig. 5 (a), we illustrate the AWCD for SFR1 when Spread Factor is between 10^−1^ and 10^−10^. The lowest AWCD is observed at Spread Factor=0.1 because of the expected higher coherence within GSOM nodes. Moreover, it enables finer analysis on data particularly when there is no prior knowledge. We eliminated the nodes having only one instance to minimize the false negatives in the training sample. Accordingly, the number of nodes varies between 300 and 920 when Spread Factor is between 10^−10^ and 10^−1^ (Fig. 5 (b)). In SFR1 analysis, we selected the GSOM map with Spread Factor=0.1 having 919 nodes. Then, we profiled each of the nodes as ‘positive/negative/ambiguous’ according to the algorithm described in Fig. 3. For instance, when a node has all unlabeled instances then it is profiled as a ‘negative node’. Accordingly, we inferred 4066 negatives.

##### GSOM clustering using SFR2:

**Fig. 6:**
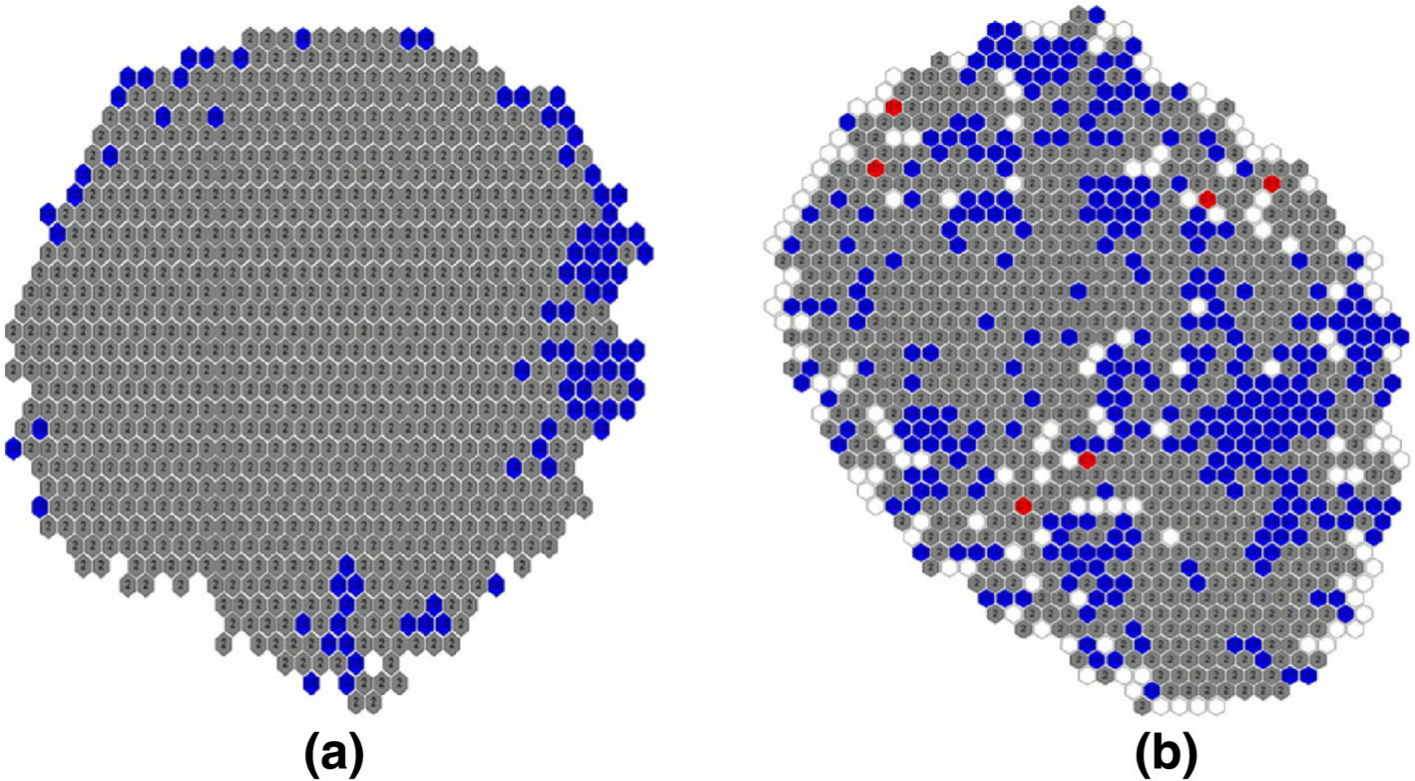
GSOM maps for DDI data: (**a**) shows the GSOM map for Similarity Feature Representation 1 (SFR1) when Spread Factor=0.1 and it contains 919 nodes; (**b**) shows the GSOM map for Similarity Feature Representation 2 (SFR2) when Spread Factor= 10^−15^ and it contains 922 nodes. The nodes shown in *blue* are the proposed negative nodes having only unlabeled instances, the nodes shown in *grey* contains both initial positives and unlabeled instances, and the nodes shown in *red* contains only initial positives

Similarly, we observed GSOM node variation for SFR2, employing 228 PCs. We selected the GSOM map with 922 nodes when Spread Factor= 10^−15^. After profiling each of the nodes as positive/negative/ambiguous, 20,099 negatives were inferred. In order to construct a reliable negative set, we filtered the common negatives captured in both approaches. As a result, only 589 common negatives (see Additional file 2) were identified. These 589 examples are the consensus negatives identified by both SFR1 and SFR2 which demonstrates a higher possibility to be served as negative examples when learning the binary classifier. (Figure 6 shows the GSOM map for SFR1 and SFR2.)

##### Binary classification:

In related work, use of balanced training sets has been shown to be beneficial for SVM classifiers [1, 6, 43]. Therefore, we propose an ensemble learning approach that integrates multiple balanced datasets for both SFRs (SFR1 and SFR2). The training sample for DDIs includes the 6036 known DDIs, extracted from DrugBank and the aforementioned 589 inferred negatives. In order to perform an ensemble learning approach, we constructed multiple balanced training sets using the training data for SFR1 and SFR2. For both SFR1 and SFR2, we constructed 10 training sets using the 589 inferred negatives and randomly selected 589 known DDIs (from the 6036 known DDIs).

We used a SVM classifier employing a polynomial kernel with a polynomial order of 2. The SVM classifier was trained using 5-fold cross validation and a suitable Regularization Parameter (C) is selected accordingly. We employed Matlab implementation for learning SVM classifier and for computing SVM posterior probability. For SFR1 and SFR2, Regularization Parameter is selected to be 10^−2^ and 10^−3^ respectively.

Table 1 shows the cross validation performance of the proposed GSOM-based PUL method, Baseline method and OCSVM for SFR1 and SFR2 using the complete balanced training dataset (589 positives + 589 negatives). It summarizes the mean performance of the 10 balanced training samples where the mean Precision, Recall, and F1-score from 5-fold cross validation are shown. There is a significant improvement in DDI prediction on Baseline and GSOM-based PUL approach when the proposed SFR2 is used though there is no significant change on OCSVM. For instance, F1-score has improved by 31.3 and 7% when using SFR2 for Baseline and GSOM-based PUL approach, respectively. On the other hand, our proposed GSOM-based PUL approach outperformed Baseline by 38.1 and 13.8% in F1-score for SFR1 and SFR2, respectively. Moreover, there are notable improvement in Precision and Recall when our proposed GSOM-based PUL approach is used.

**Table 1:** Performance assessment of the proposed GSOM-based PUL approach, Baseline and OCSVM using Similarity Feature Representation1 (SFR1) and Similarity Feature Representation2 (SFR2)

			Baseline	OCSVM	GSOM-based PUL
Cross validation	SFR1	Precision	0.628	0.584	0.951
		Recall	0.448	0.499	0.861
		F1-score	0.523	0.537	0.904
	SFR2	Precision	0.823	0.622	0.974
		Recall	0.850	0.436	0.975
		F1-score	0.836	0.511	0.974

##### Ensemble learning:

The 10 classification models of SFR1 and SFR2 obtained using our GSOM-based PUL approach are considered for DDI prediction. We employed ensemble learning aggregating the classification results of each classification model to minimize the variance of the final output. Each prediction is made by invoking a posterior probability. Finally, 20 predicted posterior probabilities are averaged to derive the final predictions.

As explained in the above section, our training set consist of 589 positives and 589 negatives. In order to assess the ensemble model, 80% of this complete balanced training set are used for training while 20% of the complete balanced training set are held out to test the overall predictive performance of the ensemble model. In Table 2, we assess the performance of the ensemble model using SFR1 and SFR2. It summarizes the mean performance of the 10 balanced training and test samples. There is no significant improvement in the ensemble model compared to the model generated using SFR2. However, the results of the ensemble model is considered for further analysis as ensemble learning has evidenced to minimize the variance of the final output [44]. We inferred 5892 potential DDIs with the greatest probability (see Additional file 3). In “Case study: clinical implications” section, we provide a case study on these predicted positive DDIs associating plausible CYPs.

**Table 2:** Performance assessment of the ensemble model for GSOM-based PUL approach for DDI prediction using Similarity Feature Representation1 (SFR1) and Similarity Feature Representation2 (SFR2)

		SFR1	SFR2	Ensemble model
Cross validation (80%)	Precision	0.960	0.968	
	Recall	0.841	0.972	
	F-measure	0.896	0.970	
Testing (20%)	Precision	0.749	0.973	0.970
	Recall	0.648	0.979	0.975
	F-measure	0.692	0.976	0.973

## Discussion

### GSOM-based positive-unlabeled learning

Our results suggest the importance of using a representative training sample in achieving better performance, as well as revealing the value of clustering methods to infer negatives; clustering clearly outperforms a random strategy for selecting negatives. Since there exists no gold standard database to capture drug pairs that do not interact with each other, the proposed GSOM-based method can be used to identify representative negatives to train a binary classifier. Moreover, GSOM is a direct approach to group similar drug pairs with no prior knowledge about the group labels. Since the number of possible drug interaction types is unknown, DDIs in GSOM nodes can be considered as sub-groups of related DDIs. Hence, the negatives can be identified in relation to the scattered pattern of known positives.

In related work, randomly selected unlabeled data has been considered as negatives or neutral DDIs. Importantly, we have shown using benchmark data, that the increasing the number of unreliable negatives in the training sample reduces the predictive performance of the binary classifier.

GSOM is capable of handling high dimensional features, and therefore it is more useful when employing the proposed pairwise drug similarity. The number of growing nodes can be controlled by its spread factor ranging between 0 and 1. Higher spread factor results in producing more coherence within nodes. However, higher coherence GSOM nodes may result in generating nodes with a single member. This approach may produce false negatives, if a node consists of only unlabeled positives. In this study, the nodes with only one instance is not considered for node profiling to minimize this error.

Selecting a suitable spread factor is challenging as we are unaware of the number of DDI types. One approach is to consider the properties of GSOM which clearly distinguish positives and negatives in choosing the most appropriate map. This approach is used when deciding the spread factor for the adapted Breast cancer data, and Iris data (see Additional file 1). However, a method to determine the spread factor is yet to be found, particularly when there is no information about the clusters. Here, we selected the GSOM maps for SFR1 and SFR2 with a relatively similar number of nodes.

Because of various types of DDIs, we noticed the scattered pattern of known positives in the GSOM map. Thus the proposed node profiling method was useful in identifying plausible negatives. The assigned positive proportion of the GSOM node is considered when profiling a node as ‘positive node/negative node/ambiguous node’. The inferred negatives were extracted from the proposed negative nodes. The results on adapted Breast Cancer and Iris data suggest the importance of the proposed approach particularly when Unlabeled Positive Proportion (UPP) is below 60% (see Additional file 1). Inferring negatives is much accurate for the datasets with lower UPPs. Since the UPP value is unknown for the DDI prediction task, the negative set has been further refined by selecting the common negatives of SFR1 and SFR2. These predicted negatives could be verified from the PubMed articles and other related resources using text mining. Text mining approaches tend to identify the co-occurrence of drug pairs in biomedical publications. But, there is no guarantee that those drug pairs would interact with each other in reality. Therefore, the drug pairs identified by text mining approaches would also require further validation to verify the prediction.

The proposed GSOM-based method can also be used in identifying representative training samples in any dataset when there is an uncertainty about the labeled data. For instance, in Breast Cancer data, the proposed GSOM-based method has extracted 236 (out of 286) more representative negatives while eliminating redundant or ambiguous instances (see UPP=0% in Additional file 1: Figure 1). This may enable the construction of efficient classification as well. Moreover, GSOM’s topology preserving nature can be employed in selecting a representative training set, particularly to resolve the imbalanced data issues if the GSOM clustering has clear separating boundaries between classes (as in Additional file 1: Figure 2). The nodes that are closer to the separation boundary may reflect close relationships to the other class. Therefore, selecting training instances away from the separation boundary can be a useful aspect to define a representative training sets. It may also improve the distinction between classes and efficient classification. Also, GSOM can be used even when there are multiple classes.

SVM is frequently used as the binary classifier in PUL and DDI prediction applications. Results on Breast Cancer Data and Iris Data demonstrate better performance when the UPP is below 50%. Since we used 6036 initial positives, predicting up to additional 6036 DDIs with higher probability is feasible. In this study, we inferred 5892 DDIs with the posterior probability above 0.995. Importantly, the proposed GSOM-based PUL approach led to achieve F1-score of 0.904 (SFR1) and 0.974 (SFR2) while in the frequently used Baseline achieved F1-score of 0.522 (SFR1) and 0.836 (SFR2). This represents approximately a 38 and 14% absolute improvement over Baseline approach using SFR1 and SFR2, respectively. On the other hand, application of OCSVM does not perform well on SFR1 and SFR2. Since the drug pairs span a large variation of DDI types, employing a representative training data for the opposing class enables the classifier to accurately distinguish between positives and negatives. Our results reveal the need for a reliable training set for achieving better performance as well as inferring reliable DDIs. Even though Liu et al. [22] achieved Specificity ranging between 0.89 and 0.97, and Sensitivity ranging between 0.13 and 0.71, our proposed approach achieved 0.97, 0.98, and 0.97 as Precision, Sensitivity/Recall, and F1-score, respectively. We attribute our performance to the strength of our method. Another approach to consider would be active learning. But, it may require a large memory and time as we are dealing with a large number of drug pairs on high dimensional space.

#### Evaluation of the proposed GSOM-based PUL approach employing INDI data

INDI is a DDI prediction tool which uses seven heterogeneous pairwise drug similarity scores based on chemical, ligands, side effects, Anatomical Therapeutic and Chemical classification, sequence similarity, distance on a protein–protein interaction network, and Gene Ontology. The proposed approach was further evaluated employing the seven heterogeneous pairwise drug similarity scores of 805 drugs used in INDI [6]. It provides the DDI labels as binary scores which are categorized as CYP related DDIs and non-CYP related DDIs for the corresponding drug pairs generated using the 805 drugs. We merged CYP related DDIs and non CYP related DDIs to construct the corresponding DDI label of each drug pair and we obtained 25103 positive DDIs. We constructed a heterogeneous feature vector similar to SFR1 with seven dimensions where the binary class labels indicate the DDI status of the corresponding drug pair.

Employing the proposed GSOM node profiling algorithm (see Fig. 3), we inferred 4352 neutral DDIs to serve as negatives for binary classifier. Table 3, summarizes the cross-validation performance assessment of DDI prediction using INDI dataset. We compared the performance of the proposed method against Baseline method and OCSVM. It is clear that the proposed GSOM-based PUL approach outperforms the other two approaches for DDI prediction using INDI data as well.

**Table 3:** Cross-Validation performance assessment of the proposed GSOM-based PUL approach, Baseline and OCSVM for DDI prediction using INDI dataset

	Baseline	OCSVM	GSOM-based PUL
Precision	0.610	0.583	0.917
Recall	0.529	0.583	0.916
F1-score	0.567	0.583	0.916

### Pairwise drug similarity

The proposed pairwise drug similarity function (SFR2) is simple yet more useful to capture finer-grained similarities as compared to the similarity metrics like Jaccard Index (JI). This is because two drug pairs with very different underlying characteristics can have the same JI. The proposed pairwise similarity function can capture the heterogeneous similarity properties between any two drugs at a more fine-grained level of representation. It can be extended to compare more than two drugs as well as in other domains where pairwise similarities are required.

Both pairwise similarity representations used in this paper demonstrate better performance when using our GSOM-based PUL approach. Therefore, aggregating classification models obtained from JI inspired pairwise similarity function (SFR1) and SFR2 enables obtaining reliable predictions. Without restricting the similarity comparison for Jaccard variants, ensemble learning using multiple distance measures like Euclidean, cosine, correlation, etc. may also increase the predictive performance.

SFR1 and SFR2 pairwise drug similarity representations used in this paper are employed separately. Concatenating SFR1 and SFR2 is another possible extension to this approach. When using SFR2, we employed PCA for dimensionality reduction. As a result, we obtained 228 PCs capturing 90% of maximum variance. Further reduction of the dimensions is not realistic as some of the pairwise drug similarity features may unique and some data are truly high dimensional. Even though GSOM clustering algorithm is capable of handling high dimensional data, preprocessing using PCA can be beneficial in improving memory and time efficiency.

In this work, we represented the pairwise drug similarity in terms of their chemical structure, disease, protein, and side effects characteristics and obtained higher performance with these four types of features. Nevertheless, integration of other types of similarity features like ligands based characteristics and 3D chemical structures may further strengthen the predictive performance and contribute to making our approach more robust. Not all predicted drug pairs will be co-administered in practice, but, deeper analysis of their individual properties may lead to derive useful drug repositioning opportunities.

### Case study: clinical implications

Cytochrome P450 (CYP) is an enzyme system found throughout many organs and tissues including the kidneys, lungs and gut however its highest concentration is in the liver. Most drugs used clinically are metabolized by the six main CYP enzymes: CYP1A2, CYP2C9, CYP2C19, CYP2D6, CYP2E1, and CYP3A4 [45–47]. Interestingly, a single drug can act as an inducer, inhibitor or a substrate of these enzymes. Knowledge in this area has expanded rapidly in recent years and a number of emerging clinically relevant interactions have been addressed [3]. Clinically significant effects usually arise when drugs which either inhibit or induce particular CYP enzyme are administered together. If one drug inhibits the metabolism of another concurrently administered drug, then this may lead to the accumulation of that second drug and possible toxicity [3, 45, 47].

#### Classify CYP-dependent and CYP-independent DDIs

DDIs can be associated to cytochrome P450 (CYP) enzyme system as well as molecular targets for drug action including receptors, ion channels and transporters or carrier molecules [2]. Most of the drug metabolism occurs via CYP enzyme system. Currently, there are over seventy (70) CYP gene families, of which three main ones (CYP1, CYP2, CYP3) are involved in drug metabolism in human liver [46]. Therefore, identification of related CYP enzymes for the predicted DDIs is considered to be significant.

In particular, clustering algorithms can be employed using features only, independently of the class labels. Hence, we extended the DDI classification task to predict plausible CYPs by revisiting the GSOM map/s (Fig. 6). According to the algorithm explained in Fig. 3, an ambiguous node contains known DDIs and unlabeled inputs. For each of the ambiguous nodes (Fig. 3; Step 4.3), we assign CYPs based on DrugBank description of already allocated positives. Such node is considered as CYP-Independent if it’s known DDIs do not have any CYP-Dependent interactions. If it has at least one known CYP-Dependent interactions, then it is considered as a CYP-Dependent node and the known CYP is also noted. In addition, the negative nodes are considered to be CYP-Independent (Fig 3; Step 4.2) as they do not contain any known DDI interactions. Accordingly, we assign CYPs for the predicted DDIs in relation to their allocated nodes on the GSOM maps.

Consequently, we classify the predicted 5892 DDIs as CYP-Dependent and CYP-Independent DDIs by revisiting the GSOM maps. We assigned CYP/s in relation to their nodes on both GSOM maps. Accordingly, we inferred 5300 CYP-Dependent DDIs (see Additional file 4) and 592 CYP-Independent DDIs (see Additional file 5). We further categorized the CYP dependent DDIs into CYP1A2, CYP2C9, CYP2C19, CYP2D6, and CYP3A4 isoforms by analyzing DrugBank description of already known DDIs at ‘ambiguous nodes’. It should also be noted that 3311 CYP-Dependent DDIs were predicted as to be associated with more than one CYP isoform. Furthermore, out of the 5300 predicted CYP-Dependent DDIs, 1083 DDIs overlap with the recently published CYP related DDIs predicted using quantitative structure-activity relationships models [48]. In the next section, we provide the clinical relevance of three predicted CYP-Dependent DDIs in relation to their predicted CYPs.

#### Clinical relevance

In this study, we inferred numerous predicted DDIs that appear to have significant clinical relevance [49]. Cardiovascular and mental health, diabetes mellitus, asthma and infection control are some of the National Health Priority Areas chosen by the Australian government due to their significant contribution to the burden of illness in Australian community [50]. In this study, we selected the predicted DDIs with a focus on medications frequently used in treating some of the common diseases: Bosentan-Abacavir, Carvedilol-Metformin and Cimetidine-Erythromycin. (It should be noted that these predictions were made for initially on unlabeled data and not mentioned in DrugBank.)

*Bosentan*, an endothelin receptor antagonist indicated for pulmonary arterial hypertension, is an inducer of CYP2C9 and a substrate for the same as well as inducer and a substrate for CYP3A4. *Abacavir*, an antiretroviral drug indicated for HIV infection, is mainly metabolised by the liver [49]. When *Abacavir* is combined with *Bosentan*, its efficacy can be significantly decreased because *Bosentan* induces CYP enzyme system thus speeding up *Abacavir* metabolism.

DDIs inferred in this study include an interaction between *Carvedilol* and *Metformin*. *Carvedilol*, non-selective beta and alpha1 receptor blocker, controls cardiac contractility and blood pressure and is widely used clinically in the management of hypertension and chronic systolic heart failure. Metformin, an oral hypoglycaemic agent used in the management of type II diabetes, targets the liver in order to reduce hepatic glucose production [49]. *Carvedilol* is a substrate for CYP2D6 and when combined with *Metformin*, the activity of CYP2D6 may be altered thus leading to loss of its therapeutic effectiveness.

Another clinically relevant DDI inferred in this study relates to an interaction between *Cimetidine* and *Erythromycin*. *Cimetidine*, a histamine H2 receptor antagonist that reduces gastric acid secretion, is used in the management of peptic ulcer disease, dyspepsia and gastro-oesophageal reflux disease while *Erythromycin* is an anti-infective that inhibits bacterial protein synthesis and also acts as an immuno-modulatory and anti-inflammatory agent [49]. Both drugs inhibit CYP3A4 which in turn leads to decrease in their metabolism and results in an enhancement of the adverse effects and toxicity.

## Conclusions

Identifying new drug-drug interactions is crucial in improving clinical care. The number of already identified drug-drug interactions on DrugBank is significantly low compared to the number of possible drug combinations. On the other hand, there is no certain database which represents the drugs that do not interact with each other. In this paper, we propose a new Positive-Unlabeled Learning approach based on Growing Self Organizing Map (GSOM) leading to identify plausible drug-drug interactions. Particularly, the proposed Positive-Unlabeled Learning approach is suitable when the number of classes is unknown or unavailable in advance. GSOM is useful in clustering drug-drug interactions based on their pairwise similarities since there are various types of drug-drug interactions. Moreover, we propose a pairwise similarity function to quantify the overlap of individual attributes of the drug feature vector. Our results reveal the importance of the proposed Positive-Unlabeled Learning approach and the proposed pairwise similarity function in identifying plausible drug-drug interactions. In addition, the proposed GSOM node labeling algorithm is used to associate cytochrome P450 isoforms for the predicted drug-drug interactions and we provide the significant clinical relevance of three pairs of the predicted drug-drug interactions. The proposed approach is a promising strategy to identify plausible drug-drug interactions and our discoveries can be used to improve clinical care as well as the research outcomes of drug development. Further, the proposed GSOM-based Positive-Unlabeled Learning approach can be applied in other applications where appropriate.

## Additional files

Additional file 1 Adapting Labeled Data for Positive-Unlabeled Learning. This file comprises the significance of the proposed method for adapted labeled data. (PDF 313 kb)

Additional file 2 The 589 inferred negatives for binary classification. This is Supplementary Table 1. (XLSX 18.1 kb)

Additional file 3 The inferred 5892 DDIs and their probabilities. This is Supplementary Table 2. (XLSX 134 kb)

Additional file 4 The inferred 5300 CYP-Dependent DDIs and associating CYPs. This is Supplementary Table 3. (XLSX 177 kb)

Additional file 5 The inferred 592 CYP-Independent DDIs. This is Supplementary Table 4. (XLSX 18.2 kb)

## Abbreviations

AWCD: Average within cluster distance
CYP: Cytochrome P450
DDI: Drug-drug interaction
GSOM: Growing self organizing map
JI: Jaccard index
OCSVM: One class support vector machine
PCA: Principal component analysis
PUL: Positive-unlabeled learning
SFR1: Similarity feature representation1
SFR2: Similarity feature representation2
SOM: Self organizing map
SVM: Support vector machine
UPP: Unlabeled positive proportion
