# Supplementary material for: Positive-Unlabeled Learning for inferring drug interactions based on heterogeneous attributes
Source: BMC Bioinformatics. 2017 Mar 1;18:140. doi: 10.1186/s12859-017-1546-7 (PMC5333429; doi:10.1186/s12859-017-1546-7)
Supplement: Additional file 1 — Adapting Labeled Data for Positive-Unlabeled Learning. This file comprises the significance of the proposed method for adapted labeled data. (PDF 313 kb) [file 12859_2017_1546_MOESM1_ESM.pdf]

Additional File 1

1 Adapting Labeled Data for Positive-Unlabeled Learning

To the best of our knowledge there exists no benchmark dataset to test Positive-Unlabeled Learning (PUL) methods. We therefore modified two existing benchmark datasets to resemble the positive and unlabeled data scenario in order to test our proposed approach.

1.1 Breast Cancer Data

The Breast cancer Wisconsin (Diagnostic) dataset is one of the popular benchmark datasets used in binary classification. It is available at the UCI machine learning repository [1]. It contains 569 instances where each of the instances is described in terms of 30 real-valued features, considering 10 main measurements of the cell nucleus. There are 212 malignant records which are considered as positives while 357 benign records are considered as negatives. Moreover, the original data has to be normalized to avoid bias on a particular feature. We normalize the features using Z-score standardization [2]. Then, 20% of the original data (113 instances) are held out for testing the method while the 80% of the data (456 instances) are used for adapting and training. In Section 1.3, we explain how we adapt labeled data for positive and unlabeled learning.

1.2 Iris Data

The Iris dataset available at the UCI machine learning repository [1] is another benchmark dataset used in classification. It includes 150 instances, 50 in each of 3 classes, Setosa, Versicolor and Verginica. Each of the instances is represented in terms of 4 attributes, considering sepal and petal measurements. These 150 instances are categorized into 3 classes as Setosa, Versicolor, and Virginica and each class has got 50 instances. In this study, we focus on classifying Setosa (50) vs Non-Setosa (Versicolor and Virginica: 100) where Setosa is considered as positives and rest of the instances are considered as negatives. As above, the original data has to be normalized to avoid bias on a particular feature. We normalize the features using standard deviation. Then, 20% of the original data (30 instances) are held out for testing the overall approach while the 80% of the data (120 instances) are used for adapting and training. In the next section (Section 1.3), we explain how we adapt labeled data for positive and unlabeled learning.

1.3 Construction of Adapted Positive and Unlabeled Datasets

Aforementioned training/adapting data is used to create PU datasets. In our experiments, 20% of the original data are held out to test the overall predictive performance while the 80% of the original data are used for developing and training the model.

In order to mimic the PU context for these data sets, randomly selected known positives and all negatives are masked as negatives in the training data. This Unlabeled Positive Proportion (UPP) is selected to vary from 0% to 90% positives in the training data. Accordingly, we constructed 10 different PU datasets by varying UPP. UPP is defined as follows:

$$Unlabeled\ Positive\ Proportion\ (UPP) = \frac{number\ of\ masked\ positives}{total\ number\ of\ positives} \quad (1)$$

For instance, when UPP is 10%, the PU dataset is constructed masking the labels of randomly selected 10% of known positives and all negatives.

2 GSOM-based PUL Approach for Adapted PU Datasets

In this section, we demonstrate the significance of the proposed PUL approach using the adapted benchmark datasets. We compared the proposed GSOM-based PUL approach against Baseline and OCSVM for varying UPP (see Section 1.3). In the GSOM-based PUL approach, we employed known positives and negatives inferred by GSOM from the unlabeled data. We employed Baseline using known positives and randomly selected negatives from the unlabeled data while OCSVM is employed using only known positives.

2.1 Adapted Breast Cancer Data:

There exist 170 positives and 286 negatives in the training set while 42 positives and 71 negatives exist in the test set. Fig 1 illustrates GSOM's capability of selecting a representative training sample for the unlabeled negative class when UPP is varying between 0% and 90%. The proposed GSOM-based PUL approach led to identify potential negatives for UPP between 0% and 50% the accuracy of the negative extraction lies above 90% when UPP is below 50%. Although the number of false negatives has gradually increased as UPP is increasing, it is significantly low compared to the number of initial unlabeled instances. In addition, Fig 2 corresponds to the correct identification of negative nodes for the adapted breast cancer data when UPP is 20%. It should also be noted that the node-112 contains one known positive and it is topologically located with other negative nodes as it has showed closer similarity with other negatives. On the other hand, node-61, is incorrectly profiled as a negative node as it has only one unlabeled positive instance. But, this error is minimized by removing nodes with only one instance.

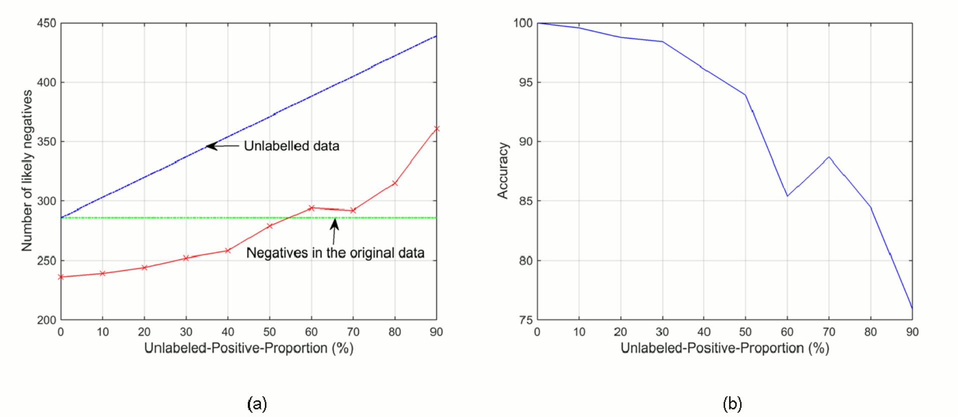

Figure 1: For Breast Cancer Data: (a) Negatives identified by the proposed GSOM-based PUL method for varying Unlabeled Positive Proportion (UPP): The dotted line shown in green represents the actual negatives which is 286 in the original training set, the dotted line shown in blue represents the total unlabeled data; (b) Accuracy of the proposed GSOM-based PUL method in inferring negatives.

Fig 3 illustrates F1-score for classifying malignant and benign instances using the proposed GSOM-based PUL approach against Baseline and OCSVM. It shows

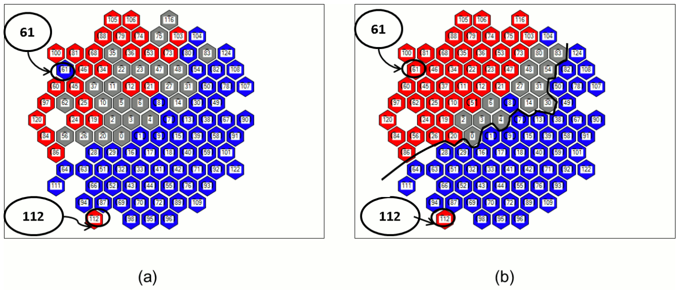

Figure 2: GSOM maps (Breast Cancer Data): (a) Profiling of GSOM nodes when Unlabeled-Positive Proportion (UPP) is 20%;(b) Profiling of GSOM nodes using original labels; The nodes shown in blue are the proposed negative nodes having only unlabeled instances, the nodes shown in grey contains both initial positives and unlabeled instances, and the nodes shown in red contains only initial positives.

the results only for UPP from 0% to 50% (UPP above 50% is ignored due to lack of adequate positive instances in the training samples). It is clear that the GSOM-based PUL approach outperforms the Baseline and OCSVM in all instances. Moreover, the final classification performance correlates strongly with the accuracy of the training set.

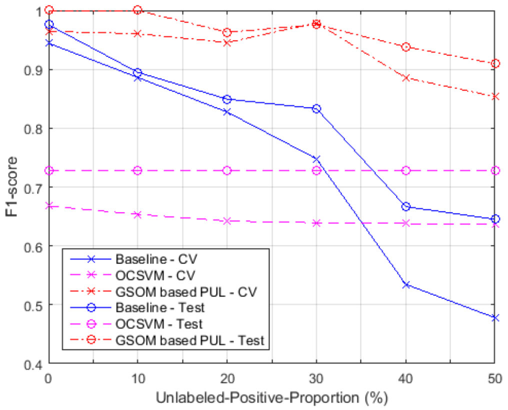

Figure 3: For Breast Cancer Data: Performance assessment of the GSOM-based PUL approach, Baseline and OCSVM for varying Unlabeled-Positive-Proportion (UPP) using Linear SVM classifier when Regularisation Parameter (C)=1. (Here we included the cross-validation (CV) and testing performance (Test) on binary classification).

2.2 Adapted Iris Data:

Fig 4 illustrates the intrinsic evaluation of the proposed GSOM-based PUL approach in identification of reliable negatives for varying UPPs between 0% and 90%. It has been correctly identified reliable negatives when UPP is below 40%. But the performance degrades as UPP is increasing. According to Fig 4, GSOM's performance of negative extraction has decreased when UPP is above 60%. Thus, predictive performance may further degrade when UPP is above 60%. Fig 5 illustrates F1-score

for classifying Setosa and Non-Setosa using the proposed GSOM-based PUL approach against Baseline and OCSVM. It shows the results only for UPP from 0% to 50% (UPP above 50% is ignored due to lack of adequate positive instances in the training samples). It is clear that the GSOM-based PUL approach outperforms the Baseline and OCSVM in all instances. Moreover, the final classification performance correlates strongly with the accuracy of the training set.

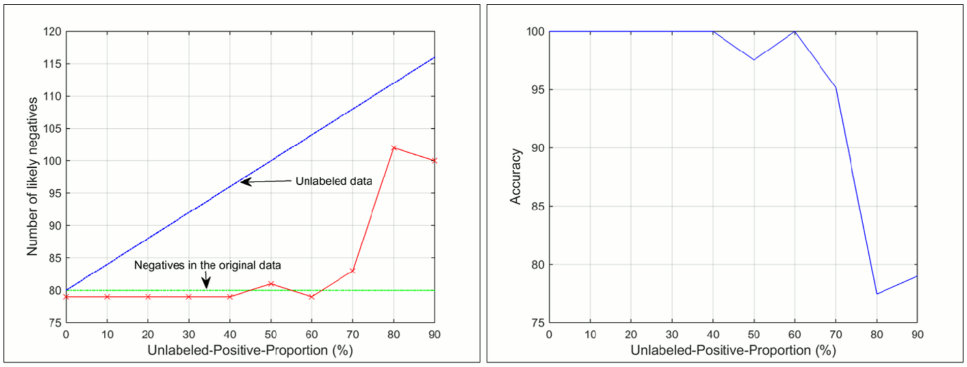

(a) (b)

Figure 4: For Iris Data: (a) Negatives identified by the proposed GSOM-based PUL method for varying Unlabeled Positive Proportion (UPP): The dotted line shown in green represents the boundary of the actual negatives which is 80 in the original training set, the dotted line shown in blue represents the boundary of the total unlabeled data; (b) Accuracy of the proposed GSOM-based PUL method in inferring negatives.

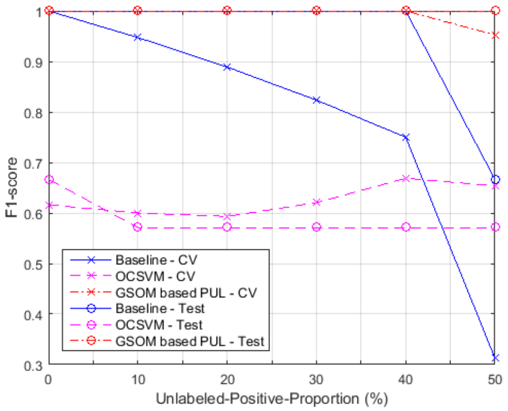

Figure 5: For Iris Data: Performance assessment of the GSOM-based PUL approach, Baseline and OCSVM for varying Unlabeled-Positive-Proportion (UPP) using Linear SVM classifier when Regularisation Parameter (C)=1. (Here we included the cross-validation (CV) and testing performance (Test) on binary classification.)

Author details

References

1. Asuncion, A., Newman, D.: UCI machine learning repository (2007)
2. Larose, D.T.: Discovering Knowledge in Data: an Introduction to Data Mining. John Wiley & Sons (2014)
